# Evaluating performance of covariate-constrained randomization (CCR) techniques under misspecification of cluster-level variables in cluster-randomized trials

**DOI:** 10.1016/j.conctc.2021.100754

**Published:** 2021-02-16

**Authors:** Madeleine Organ, S. Darius Tandon, Alicia Diebold, Jessica K. Johnson, Chen Yeh, Jody D. Ciolino

**Affiliations:** a Department of Preventive Medicine, Division of Biostatistics, Feinberg School of Medicine, USA; b Department of Preventive Medicine, Division of Biostatistics, Feinberg School of Medicine, Northwestern University, Chicago, IL, USA; c Center for Community Health, Institute for Public Health and Medicine, Feinberg School of Medicine, Northwestern University, Chicago, IL, USA

**Keywords:** Cluster-randomized trials, Covariate-constrained randomization, Simple randomization

## Abstract

Covariate constrained randomization (CCR) is a method of controlling imbalance in important baseline covariates in cluster-randomized trials (CRT). We use simulated CRTs to investigate the performance (control of imbalance) of CCR relative to simple randomization (SR) under conditions of misspecification of the cluster-level variable used in the CCR algorithm.

We use data from a Patient-Centered Outcomes Research Institute (PCORI)-funded CRT evaluating the Mothers and Babies (MB) intervention (AD-1507-31,473). CCR methodology was used in the MB study to control imbalance in, among other baseline variables, the percent minority (i.e., non-White) participants at each study site. Simulation schemes explored variation in degree of misspecification in the baseline covariate of interest, and include correct report, observed misspecification, and a range of simulated misspecification for intervals within and beyond that observed in the MB study. We also consider three within-site sample size scenarios: that observed in the MB study, small (mean 10) and large (mean 50). Simulations at every level of baseline covariate misspecification suggest that use of the CCR strategy provides between-arm imbalance that is simultaneously lower and less variable, on average, than that produced from the SR strategy. We find that the gains to using CCR over SR are nearly twice as high with accurate reporting (Δ = −5.33) compared to the observed study-level misspecification (Δ = −3.03). Although CCR still outperforms SR as the level of misspecification increases, the gains to using CCR over SR decrease; thus, every effort should still be made to obtain high-quality baseline data.

## 1 Introduction

In cluster-randomized trials (CRTs), covariate-constrained randomization (CCR) tends to increase efficiency over simple randomization (SR) in controlling imbalance in baseline characteristics across arms [1]. CCR methods require baseline data collection on important cluster-level variables to control imbalance at the cluster level, which acts as a surrogate for the ultimate goal of achieving adequate control of imbalance at the participant level. Thus, if a site inaccurately reports baseline covariates used to control imbalance in the CCR algorithm, one would expect poor control over participant-level imbalance, with performance potentially reverting back to that of SR. This simulation study evaluates the methods of randomization for a Patient-Centered Outcomes Research Institute (PCORI)-funded study involving the Mothers and Babies (MB) intervention (AD-1507-31,473), which targets prevention and reduction of postpartum depression among low-income women [2,3].

Complex randomization schemes like CCR aim to reduce imbalance in important covariates between treatment arms. Imbalance makes it difficult to determine whether observed differences between arms is attributable to intervention, thereby limiting the ability to draw thereby causal inference and negating one of the strengths of randomized trials [4, [5], [6]]. In addition to the capabilities of other complex forms of randomization for CRTs (restricted randomization, stratified/block randomization, or matching, for example), CCR contributes feasibility when the number of constraining variables is large or the constraining variables are continuous [5]. Previous literature suggests that CCR methods are effective in avoiding serious between-arm imbalance and provide the largest gains over simple randomization when the number of clusters is small [7]. However, to our knowledge, there is no literature examining the performance of CCR relative to other randomization methods when the baseline, cluster-level covariates used in the algorithm do not accurately represent individual-level data obtained in the study.

The motivating study is a covariate-constrained CRT in which home visiting program sites were randomized to one of three study arms varying the delivery of the MB program [3]. For details on the motivation, study design, and analysis plan, see Jensen et al. [3]. Briefly, the group-based MB program has demonstrated efficacy in both prevention and reduction of postpartum depression and its symptoms among low-income women; the motivating study aims to evaluate the efficacy of the MB program when delivered by professionals versus paraprofessionals [2,[8], [9], [10], [11], [12]].

**Fig. 1:**
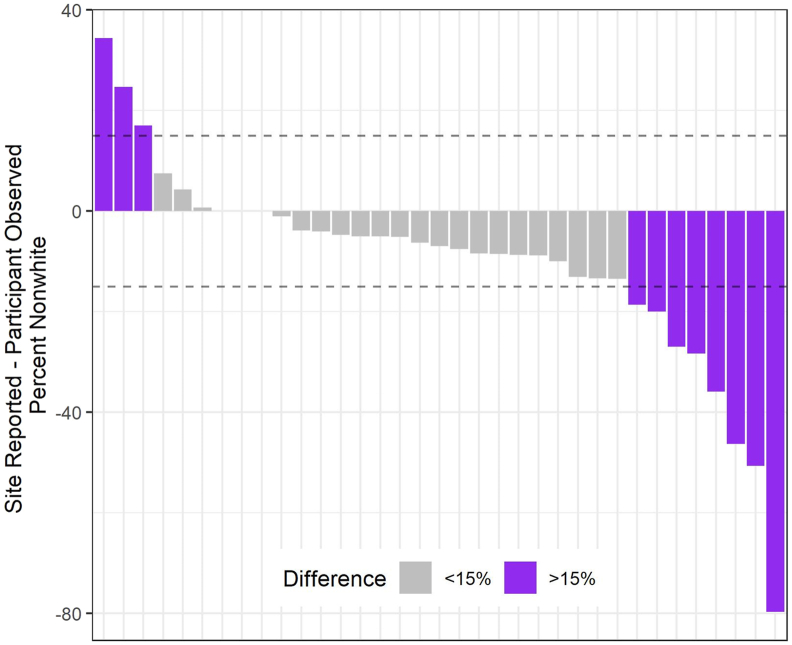
Misspecification of site-level percent minority, MB study.

Among other cluster-level baseline variables, the motivating study used the CCR algorithm to control imbalance in percent minority (i.e., non-White) clients at each site. As shown in Fig. 1, the extent to which the site-reported percent minority matched that of the observed study participants varied; we hereafter refer to this discrepancy as “misspecification.” As described in Table 1, 74% of sites under-reported their percent of minority clients; of these, sites reported an average of nearly 18 percentage points less than the observed, participant-level percent minority. This large misspecification motivated this simulation study, which aims to evaluate the performance of CCR relative to SR under similar misspecification scenarios. We explore variation in (1) the severity of misspecification, and (2) the number of participants from each site to determine the extent to which CCR controlled between-arm imbalance more efficiently than SR.

**Table 1:** Misspecification of site-level percent minority, MB study.

	All Sites		Extreme Δ Sites	
Reporting Category	N (%)	Average Δ	N (%)	Average Δ
Under Reported	26 (74%)	−16.93	8 (31%)	−38.31
Over Reported	6 (17%)	14.77	3 (50%)	25.36
Correctly Reported	3 (9%)	0.00		

*Notes:* Fig. 1 shows the extent of site-level misreporting of percent minority participants at baseline. Underreporting the percent of minority participants was much more common than overreporting, and is seen in 26 (74%) sites; among these, the average level of misspecification was 16.9 percentage points, but among the 8 sites (31% of underreporting sites) with extreme misspecification (>15 percentage points misspecification) it was 38.3 percentage points. By contrast, 6 (17%) sites overreported the percent of minority participants by an average misspecification of 14.5 percentage points; the 3 sites with extreme misspecification averaged a 25.3 percentage point deviation from their baseline reported value.

## 2 Methods

To evaluate general performance of CCR in comparison to SR methods, we considered a simplified trial design involving equal allocation into only two treatment arms. We used data from the 35 active sites in the MB study, but we allocated 17 to each arm in these simulations to ensure equal numbers of sites across arms in the simulated CRTs. The general simulation procedure is as follows and uses conventions in the CRT literature [1,4,6]:

1. Enumerate a large subset (N = 100,000) of possible allocation schemes ensuring equal (17:17) allocation.

2. Evaluate the (im)balance for the variable of interest, site-level reported percent minority, of each allocation scheme, and save a smaller subset (n = 0.1*N) of potential allocations representing the CCR and SR scenarios.
a. CCR: Save the set of potential allocations if the imbalance is acceptable according to a pre-specified criterion, defined here as the lower 10th percentile of the measure of between-arm imbalance ($D=\left|mean_{1}-mean_{2}\right|$) for all possible allocation schemes [4,6]. Here, $mean_{i}$ represents the mean percent minority for all sites in group i,i∈{1,2}.
b. SR: Save a randomly selected set of possible allocations.
3. Simulate participant-level percent minority by sampling from a binomial distribution, using variations of *N* and *p*, where *p* varies as described below; Table 2 summarizes all simulation scenarios explored.

**Table 2:** Simulation schemes: binomial distribution parameters.

Scheme	Description	N	p
**Accurate Reporting**	**1** Accurate Reporting, Site-Level N	Site-level N	Site-reported Percent Minority
	**1a** Accurate Reporting, Small N	N~N(10,5)	Site-reported Percent Minority
	**1b** Accurate Reporting, Large N	N~N(50,25)	Site-reported Percent Minority
**Study-Level Misspecification**	**2** Misspecified Reporting, Site-Level N	Site-level N	Observed Participant Percent Minority
	**2a** Misspecified Reporting, Small N	N~N(10,5)	Observed Participant Percent Minority
	**2b** Misspecified Reporting, Large N	N~N(50,25)	Observed Participant Percent Minority
**Simulated Misspecification, increasing in increments**	**3** 0–5% Misspecification, Site-Level N	Site-level N	p~UNIF(0,5)
	**3a** 0–5% Misspecification, Small N	N~N(10,5)	p~UNIF(0,5)
	**3b** 0–5% Misspecification, Large N	N~N(50,25)	p~UNIF(0,5)
	**4** 5–10% Misspecification, Site-Level N	Site-level N	p~UNIF(5,10)
	**4a** 5–10% Misspecification, Small N	N~N(10,5)	p~UNIF(5,10)
	**4b** 5–10% Misspecification, Large N	N~N(50,25)	p~UNIF(5,10)
	**5** 10–15% Misspecification, Site-Level N	Site-level N	p~UNIF(10,15)
	**5a** 10–15% Misspecification, Small N	N~N(10,5)	p~UNIF(10,15)
	**5b** 10–15% Misspecification, Large N	N~N(50,25)	p~UNIF(10,15)
	**6** 15–20% Misspecification, Site-Level N	Site-level N	p~UNIF(15,20)
	**6a** 15–20% Misspecification, Small N	N~N(10,5)	p~UNIF(15,20)
	**6b** 15–20% Misspecification, Large N	N~N(50,25)	p~UNIF(15,20)
	**7** 20–30% Misspecification, Site-Level N	Site-level N	p~UNIF(20,30)
	**7a** 20–30% Misspecification, Small N	N~N(10,5)	p~UNIF(20,30)
	**7b** 20–30% Misspecification, Large N	N~N(50,25)	p~UNIF(20,30)
**Simulated Misspecification, accumulated range**	**8** 0–20% Misspecification, Site-Level N	Site-level N	p~UNIF(0,20)
	**8a** 0–20% Misspecification, Small N	N~N(10,5)	p~UNIF(0,20)
	**8b** 0–20% Misspecification, Large N	N~N(50,25)	p~UNIF(0,20)
	**9** 0–30% Misspecification, Site-Level N	Site-level N	p~UNIF(0,30)
	**9a** 0–30% Misspecification, Small N	N~N(10,5)	p~UNIF(0,30)
	**9b** 0–30% Misspecification, Large N	N~N(50,25)	p~UNIF(0,30)

a. Accurate site-reported percent minority. The expected value of the percent minority of participants enrolled in the trial is equal to that reported for each site at study baseline.

b. Study-level misspecified site-reported percent minority. The expected value of the percent minority of participants enrolled in the trial is equal to that which we observed in the MB study; most sites misspecified the percent minority.

c. Simulated misspecification in increasing increments. Site-reported *p* perturbed by a simulated misspecification amount, where misspecification increases across simulations by small increments.

d. Simulated misspecification over an accumulated range. Site-reported *p* perturbed by a simulated misspecification amount, where misspecification is accumulated over a wide range (that includes zero).

Although we observe that most sites underreport the site-level percent minority, for this more general simulation study, we allowed for equal probability of over and underreported values. Boundaries of 0 and 1 (=0% and 100% percent minority, respectively) were enforced if the perturbation resulted in a simulated percent minority that exceeded the plausible range.

The magnitude of simulated misspecification levels is informed by the degree observed in the MB study, which represents a plausible degree of error in clinical trials, but also includes higher levels of misspecification designed to identify potential limits of the CCR algorithm. Analysis of these simulations primarily involve descriptive statistics comparing the distribution of simulated participant level imbalance ($D^{sim}=\left|Percent\;of\;Simulated\;Minority\;Participants,Arm\;1-Percent\;of\;Simulated\;Minority\;Participants,\;Arm\;2\right|)$ for the CCR and SR randomization schemes. Let $D_{CCR}^{sim}$ = the $D^{sim}$computed for CCR allocations and $D_{SR}^{sim}$represent the $D^{sim}$computed for SR allocations; we compute the mean $D^{sim}$ for each randomization type (i.e., $\bar{D_{\mathrm{CCR}}^{\mathrm{sim}}}$**and** $\bar{D_{\mathrm{CCR}}^{\mathrm{sim}}}$)and the difference between them (Δ) to measure the improvement (i.e., reduction in imbalance) of the average CCR allocation compared to the average SR allocation.

We use the worst-performing (highest imbalance) value of *D* in the correctly specified CCR scenarios as a benchmark for comparing, for a given site-level sample size construction, simulations with varying levels of misspecification. That is, we calculate the proportion of allocations with worse performance than this reference point (max $D_{CCR}^{sim}$ ) for (a) CCR allocations with misspecification, (b) SR allocations with no misspecification, and (c) SR allocations with misspecification in order to assess the gains to using CCR with accurate reporting.

## 3 Results

**Fig. 2:**
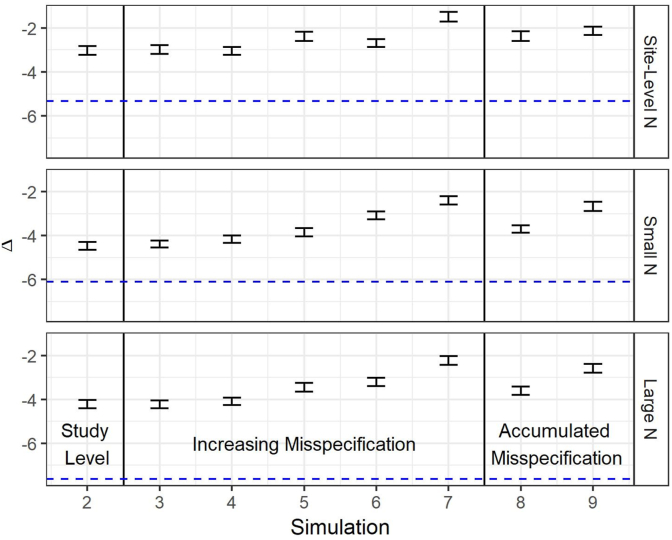
Simulation results: mean difference (Δ, 95% CI) in simulated participant between-arm imbalance $(D^{sim}$), relative to simulation with no misspecification.

Fig. 2 illustrates the difference (Δ, 95% CI) in simulated imbalance ($D^{sim}$) between randomization types under each simulation scenario; Table 3 also summarizes these results. The dashed reference line indicates the point estimate for Δ under the assumption of accurately reported data; the difference (i.e., improvement) in average imbalance (Δ) between CCR and SR is larger in magnitude than those observed in simulations involving misspecification (as Δ is computed as $D_{CCR}^{sim}-\;D_{SR}^{sim}$, negative values indicate that CCR outperforms SR). By comparison, the gains to using CCR are reduced in simulations using study-level misspecification (refer to Table 3 for Δ observed in Simulation 1 and Simulation 2; −5.33 and −3.03, respectively). As the simulated misspecification increases incrementally in simulations 3–7, the difference in average performance between CCR and SR shrinks but stays above 1.5% points in

**Table 3:** Simulation results summary: simulated individual proportion minority.

Scheme	Description	$\bar{D_{\mathrm{CCR}}^{\mathrm{sim}}}\;\left(\mathrm{se}\right)$	$\bar{D_{\mathrm{SR}}^{\mathrm{sim}}}\;\left(\mathrm{se}\right)$	Δ	95% CI for Δ	$\max\left(D_{\mathrm{CCR}}^{\mathrm{sim}\_i}\right)$	$D_{\mathrm{CCR}}>\max\left(D_{\mathrm{CCR}}^{\mathrm{sim}\_i}\right)$	$D_{\mathrm{SR}}>\max\left(D_{\mathrm{CCR}}^{\mathrm{sim}\_i}\right)$
		(1)	(2)	(3)	(4)	(5)	(6)	(7)
**Accurate Reporting**	**1** Accurate Reporting, Site-Level N	6.54 (0.05)	11.87 (0.09)	−5.33	[-5.52, −5.13]	29.41	0%	4.58%
	**1a** Accurate Reporting, Small N	4.78 (0.04)	10.88 (0.08)	−6.10	[-6.28, −5.92]	22.11	0%	10.65%
	**1b** Accurate Reporting, Large N	3.42 (0.02)	11.04 (0.08)	−7.61	[-7.78, −7.44]	14.58	0%	29.69%
**Study-Level Misspecification**	**2** Misspecified Reporting, Site-Level N	8.22 (0.06)	11.25 (0.08)	−3.03	[-3.23, −2.83]	29.41	0.10%	3.35%
	**2a** Misspecified Reporting, Small N	5.80 (0.04)	10.27 (0.08)	−4.46	[-4.63, −4.29]	22.11	0.19%	8.38%
	**2b** Misspecified Reporting, Large N	6.40 (0.05)	10.61 (0.08)	−4.21	[-4.40, −4.03]	14.58	6.44%	27.35%
**Simulated Misspecification, increasing in increments**	**3** 0–5% Misspecification, Site-Level N	8.05 (0.06)	11.03 (0.08)	−2.98	[-3.17, −2.78]	29.41	0.07%	2.93%
	**3a** 0–5% Misspecification, Small N	5.60 (0.04)	9.98 (0.07)	−4.38	[-4.55, −4.22]	22.11	0.04%	7.88%
	**3b** 0–5% Misspecification, Large N	6.11 (0.05)	10.34 (0.08)	−4.23	[-4.40, −4.05]	14.58	5.37%	26.20%
	**4** 5–10% Misspecification, Site-Level N	7.40 (0.05)	10.44 (0.08)	−3.04	[-3.23, −2.86]	29.41	0.04%	2.06%
	**4a** 5–10% Misspecification, Small N	5.86 (0.04)	10.02 (0.07)	−4.16	[-4.33, −3.99]	22.11	0.22%	7.78%
	**4b** 5–10% Misspecification, Large N	6.17 (0.05)	10.26 (0.08)	−4.09	[-4.27, −3.92]	14.58	5.43%	25.89%
	**5** 10–15% Misspecification, Site-Level N	9.29 (0.07)	11.67 (0.09)	−2.38	[-2.59, −2.17]	29.41	0.37%	3.97%
	**5a** 10–15% Misspecification, Small N	6.94 (0.05)	10.78 (0.08)	−3.84	[-4.03, −3.66]	22.11	0.84%	10.07%
	**5b** 10–15% Misspecification, Large N	7.57 (0.06)	11.02 (0.08)	−3.45	[-3.65, −3.25]	14.58	12.55%	29.65%
	**6** 15–20% Misspecification, Site-Level N	7.55 (0.06)	10.23 (0.08)	−2.69	[-2.87, −2.50]	29.41	0.04%	1.81%
	**6a** 15–20% Misspecification, Small N	6.88 (0.05)	9.96 (0.07)	−3.08	[-3.25, −2.90]	22.11	0.78%	7.58%
	**6b** 15–20% Misspecification, Large N	7.28 (0.05)	10.48 (0.08)	−3.20	[-3.39, −3.02]	14.58	10.94%	26.81%
	**7** 20–30% Misspecification, Site-Level N	10.31 (0.07)	11.80 (0.09)	−1.50	[-1.72, −1.28]	29.41	1.06%	4.20%
	**7a** 20–30% Misspecification, Small N	8.00 (0.06)	10.40 (0.08)	−2.40	[-2.59, −2.20]	22.11	2.47%	8.99%
	**7b** 20–30% Misspecification, Large N	8.34 (0.06)	10.57 (0.08)	−2.23	[-2.42, −2.03]	14.58	15.97%	27.61%
**Simulated Misspecification, accumulated range**	**8** 0–20% Misspecification, Site-Level N	9.78 (0.07)	12.15 (0.09)	−2.38	[-2.60, −2.16]	29.41	0.77%	4.85%
	**8a** 0–20% Misspecification, Small N	6.15 (0.05)	9.85 (0.07)	−3.70	[-3.87, −3.53]	22.11	0.25%	6.99%
	**8b** 0–20% Misspecification, Large N	7.11 (0.05)	10.72 (0.08)	−3.61	[-3.80, −3.43]	14.58	10.15%	27.76%
	**9** 0–30% Misspecification, Site-Level N	8.13 (0.06)	10.25 (0.08)	−2.13	[-2.31, −1.94]	29.41	0.04%	1.85%
	**9a** 0–30% Misspecification, Small N	8.71 (0.06)	11.37 (0.08)	−2.66	[-2.87, −2.45]	22.11	3.84%	12.00%
	**9b** 0–30% Misspecification, Large N	8.24 (0.06)	10.82 (0.08)	−2.58	[-2.78, −2.38]	14.58	16.33%	28.95%

magnitude, even in the case of highly misspecified (simulation 7, percent minority data misspecified by 20–30%).

*Notes:* In column 3, Δis computed as $\Delta \;=\bar{D_{CCR}}-\;\bar{D_{SR}}$ , and represents the average benefit of CCR allocations over SR allocations; increased magnitude represents increased benefit. In column 5, $max\left(D_{CCR}^{sim\_i}\right)$ is the maximum D for all CCR allocations under simulation scheme 1 (i.e, with no misspecification) with the corresponding sample size configuration; it represents the performance of the worst (greatest imbalance) CCR allocation when *no* misspecification is present, for each sample size. Columns 6 and 7 give the percent of allocations that perform worse than this upper bound of CCR performance when there is misspecified CCR (column 6), SR with no misspecification (column 7, rows 1–3), and SR with misspecification (remainder of column 7) Thus, the first three rows in column (6) are 0% by construction.

*Notes*: Fig. 2 presents the 95% confidence intervals for the difference in simulated participant between-arm imbalance (Δ) between CCR and SR randomization by misspecification level. The blue dashed line is given as a reference for the point estimate for Δ when there is no misspecification (i.e., Simulation 1). Negative estimates for Δ indicate that CCR performs better than SR. The Δ measures generally increase as misspecification increases, but still remain negative (i.e., CCR continues to outperform SR, even as misspecification increases).

Simulations 8 and 9, which use accumulated misspecification (i.e., a range from zero to the upper bound, rather than a narrow window 5 or 10 percentage points less than the upper bound) perform similarly to the simulations with the corresponding upper bound (simulations 6 and 7, respectively). The improvement of CCR over SR is similar for the Small N and Large N simulations. The Δ values for simulations with site-level N are smaller than the corresponding Δ for both small N and large N simulations, with the small N Δ values generally nominally larger than those of the large N simulations. The results for the accumulated misspecification simulations (simulations 8 and 9) are similar to the incremental simulations with the corresponding upper bound (simulations 6 and 7, respectively) with respect to the difference (Δ) in $D^{sim}$ values, although there is some difference in the magnitude of the $D^{sim}$ values themselves for the site-level N simulation. Additionally, there is a small but consistent reduction in standard error of the mean $D^{sim}$ estimates in the CCR simulations compared to the SR simulations, suggesting that an advantage of CCR beyond lower between-arm imbalance is reduced variation in the range of $D^{sim}$ values for possible allocations.

**Fig. 3:**
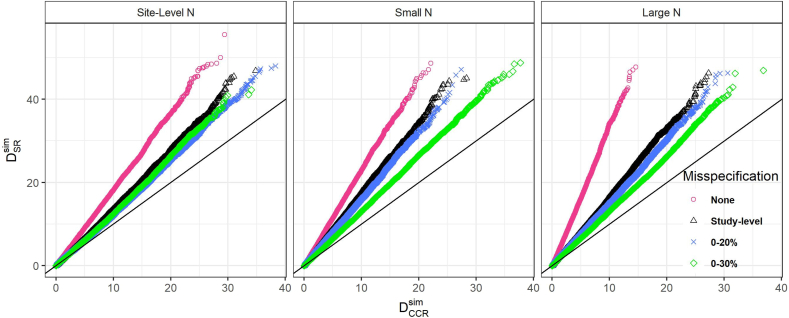
Q-Q Plots for comparing CCR and SR performance: imbalance in simulated participant percent minority by randomization type.

*Notes:* Fig. 3 plots the distribution of the $D^{sim}$ values from CCR against those of SR allocations for study-level and large-range simulated misspecification. A reference line with slope equal to one is provided and represents equivalent performance of CCR and SR; slopes greater than one, as seen here, represent improved performance in CCR relative to SR. Particularly for the Small N and Large N simulations, there is visual evidence that performance of CCR approaches – though does not reach – that of SR as misspecification increases.

The Q-Q plots in Fig. 3 show the relationship between CCR and SR allocations under conditions of accurate reporting (pink) study-level misspecification (black), and the simulations with accumulated misspecification (green, 0–20%, and blue, 0–30%). A line with a slope of 1 is given as a reference. Simulations for which points fall on this line would suggest no difference in the performance of CCR relative to SR (as the distribution of $D_{CCR}^{sim}$ would be the same as that of $D_{SR}^{sim}$). Notably, with any level of misspecification in the baseline parameters, the distribution of $D_{CCR}^{sim}$ becomes more similar to that of $D_{SR}^{sim}$; however, even with levels of misspecification equal to or exceeding those seen in this study, the deviation of the Q-Q line from the reference line indicates that CCR is still an improvement over SR with respect to controlling imbalance (*D*) across arms. For the simulations involving within-site sample sizes similar to those observed in the study data, the performance of the CCR algorithm relative to SR is similar, as evidenced by the overlap of the Q-Q points, even as simulated misspecification increases. However, for both the small N simulation and the large (and highly variable) N simulations, the figure suggests that performance of the CCR algorithm deteriorates relative to SR as misspecification increases.

The results of the benchmark comparison for an upper bound of worst performance in correctly specified analysis are presented in columns 5–7 of Table 3; column 5 gives the benchmark values, column 6 gives the percent of CCR allocations with worse performance for each misspecification level, and column 7 gives the percent of SR allocations with worse performance (rows 1–3 provide the comparison for SR allocations with no misspecification, and all remaining rows provide the comparison for SR allocations with increasing misspecification). An immediate finding of this analysis is that the proportion of allocations that performed worse than our benchmark is much higher for the large N simulation than either the small N or site-level N simulations. This finding could be partially explained by the remarkably good performance of the large-N, accurately reported CCR simulation (1b), which has a maximum simulated imbalance measure of 14.58, roughly half the size of the imbalance for the worst-performing CCR allocation with study-level within-site sample size (simulation 1, $max\left(D_{CCR}^{sim}\right)$ = 29.41). For site-level and small N simulations, we find that the proportion of CCR allocations under conditions of misspecification performing worse than the benchmark is less than 1% for most levels of simulated misspecification. For large-N simulations, the proportion of lower-performing CCR allocations is modest when misspecification is fairly small but increases for misspecification levels above 10%.

We find that the percentage of SR allocations with $D^{sim}$ greater than the benchmark at the respective sample size is much greater (4.58% with site-level *N* and no misspecification) than the corresponding percentage of CCR allocations, and that there is similarly an increase in this measure for large N simulations (29.69% under the assumption of no misspecification). Because the SR algorithm does not rely on any pre-specified cluster-level data, we expect no difference in this measure of performance for the SR allocations under accurate reporting (column 7, rows 1–3) vs. misspecified reporting (all other rows). Interestingly, we find a larger percent of SR allocations under no misspecifications surpassing our benchmark than SR allocations under conditions of misspecification.

## 4 Discussion

This simulation study contributes to the CCR literature by evaluating CCR performance under practical, rather than ideal, conditions: in real-world clinical trials, there may be a discrepancy between the reported baseline site-level covariates used to control imbalance in study arms and the site's observed data at the participant level. The various simulation schemes used here explore a range of misspecification, including intervals within and beyond the range of misspecification observed in the MB study. We find that at every level of misspecification, CCR continues to outperform SR by providing between-arm imbalance that is both lower in magnitude and less variable. The gains to using CCR over SR persist, even up to a discrepancy of 30 percentage points between the reported and observed values. However, the gains to using CCR over SR decrease as the misspecification increases in severity. These findings are robust to changes in the within-site sample size, for both simulated large and small within-site samples.

Our findings are consistent with previous literature and suggest that using the CCR strategy provides between-arm imbalance that is simultaneously smaller and less variable, on average, than that produced from the SR strategy [1,4,6,13,14]. Point estimates for the mean imbalance among simulated participants (mean $D^{sim}$ ) with corresponding standard deviations are shown in supplemental figure A1 and indicate that the measures of variance of imbalance for CCR allocations are smaller than those for the SR allocations at every level of simulated misspecification. Even without the higher levels of between-arm imbalance for SR, the increased variability for between-arm imbalance among SR allocation schemes may motivate use of CCR. In the applied context of designing a clinical trial that will implement only one randomization scheme, it is sensible to choose a strategy with minimal variability in between-arm imbalance to avoid implementing an allocation scheme with a high level of baseline imbalance simply due to chance.

There are several notable limitations of this study. First, the simulations draw many times from the set of once-randomized sample size (which determines simulation parameter *N*) or misspecification (which, along with the baseline site-reported value, contributes to simulation parameter *p*) values. If the findings here are sensitive to the simulated parameter values, an additional layer of simulations – over a range of possible binomial parameter values – may better capture the relationship between the degree of misspecification and the performance of each randomization algorithm. We also only consider a fixed number of clusters in this study (35, as in the MB study) and equal allocation of clusters into each treatment arm. Previous literature has demonstrated the advantage of CCR when the number of clusters is small [7]; it is plausible that a study with fewer clusters or unequal allocation to treatment arms may demonstrate different sensitivity to misspecification than that found here. Additionally, although we compare CCR only to SR, we acknowledge that other randomization methods, such as stratification, are commonly used. A disadvantage of using stratification in studies such as the motivating example is the requirement to use a somewhat arbitrary threshold to categorize sites based on any continuously-measured covariate (here, the percent of non-White participants). Future research could pursue simulations with variation in this threshold parameter. Stratification for the motivating study had the potential for very small strata (including cell counts of zero), making it nearly impossible to restrict randomization to control imbalance using this method. Thus, we chose to focus simulations on the situations most akin and generalizable to studies such as the motivating one.

There is great potential for follow-up analyses to continue this investigation of the relationship between the degree of site-level reporting accuracy and the performance of CCR relative to SR. Even simulations with deviations from the true percent minority up to 30 percentage points suggest the improved performance of CCR over SR, but the upper limit of reporting inaccuracy for which CCR continues to outperform SR has not yet been determined. For simplicity, the CCR algorithm implemented here considers only one baseline covariate to control imbalance in the treatment arms and is subject to potential misspecification. However, an advantage of the CCR algorithm is its ability to control imbalance in many baseline covariates simultaneously; misspecification in multiple covariates has the potential either to magnify the improvement of CCR over SR, or to mitigate the difference between the two methods. We leave the exploration of the ways that varying degrees of misspecification in multiple variables impacts randomization performance to future research, as it likely depends on the magnitude and directionality of the imbalance in multiple variables, and the varying degrees of correlation between them.

Despite these limitations, the findings from these simulations have implications for CRT design and conduct as they suggest CCR algorithms merit the added programming and logistical efforts they require; CCR continues to carry a benefit over simpler randomization methods in the presence of poor data quality for covariates included in the algorithm. These results do also highlight the importance of quality data capture prior to randomization for the cluster-level covariates used in the CCR algorithm. We find that CCR is more efficient than SR in spite of misspecification in the cluster-level variables, but that the gains to CCR increase (even doubling in magnitude) with accurate specification of the relevant covariates. The nature of the simulations implies that there will be some individual-level variation about the reported site-level parameter, even if the simulation is centered around an accurate measure. Additionally, we find a slight advantage (i.e., reduced between-arm simulated participant imbalance with CCR) for both the small N or large N simulations, relative to those taking study-level within-site sample sizes. Possibly the advantage of the large N simulation is size itself, while the small N simulation has the advantage of a small variance by construction.

While covariate-adjustment in the design of CRTs can control the imbalance between study arms at baseline, adjustment in analysis of CRTs is often necessary. While not the focus of the present manuscript, we note that even seemingly trivial levels of imbalance in important baseline covariates may lead to unsound inferences if analyses fail to adjust for these covariates [15,16]. In the CRT context, Li et al. find that relative to SR, CCR reduces the standard error of the estimate of the treatment effect in an unadjusted F-test, and although adjusted analyses result in greater power (for the same type I error rate) than the correspondingly-randomized unadjusted analyses, the improvement in power of CCR over SR in adjusted analyses is small [6]. While this implies that adjustment in analyses may alleviate many of the issues that CCR attempts to prevent in the design, it is impossible to adjust for all covariates; as stated in Lin (2015), “the pursuit of balance can be viewed as a low-cost insurance policy against the likelihood of extreme imbalances, albeit the chance of such imbalances occurring might be low” [13]. As CCR restricts the allocation space to a smaller set of better-balanced allocations, it offers the advantage of an effective upper bound (at the 10th percentile, in this case) for imbalance, based on the baseline, site-level covariates. The results of the simulations of this study suggest that this advantage of the CCR methodology holds even when the baseline covariates are misspecified.

## Trial registration

This trial is registered on ClinicalTrials.gov (Initial post: December 1, 2016; identifier: NCT02979444).

## Declaration of competing interest

We wish to confirm that there are no known conflicts of interest associated with this publication and there has been no significant financial support for this work that could have influenced its outcome.
