# Supplementary material for: Evaluating performance of covariate-constrained randomization (CCR) techniques under misspecification of cluster-level variables in cluster-randomized trials
Source: Contemp Clin Trials Commun. 2021 Feb 16;22:100754. doi: 10.1016/j.conctc.2021.100754 (PMC7941091; doi:10.1016/j.conctc.2021.100754)
Supplement: Multimedia component 1 [file mmc1.docx]

**Figure A1.** Simulation Results: $D^{sim}$ Mean (se) for simulated individual differences in proportion minority, by randomization type


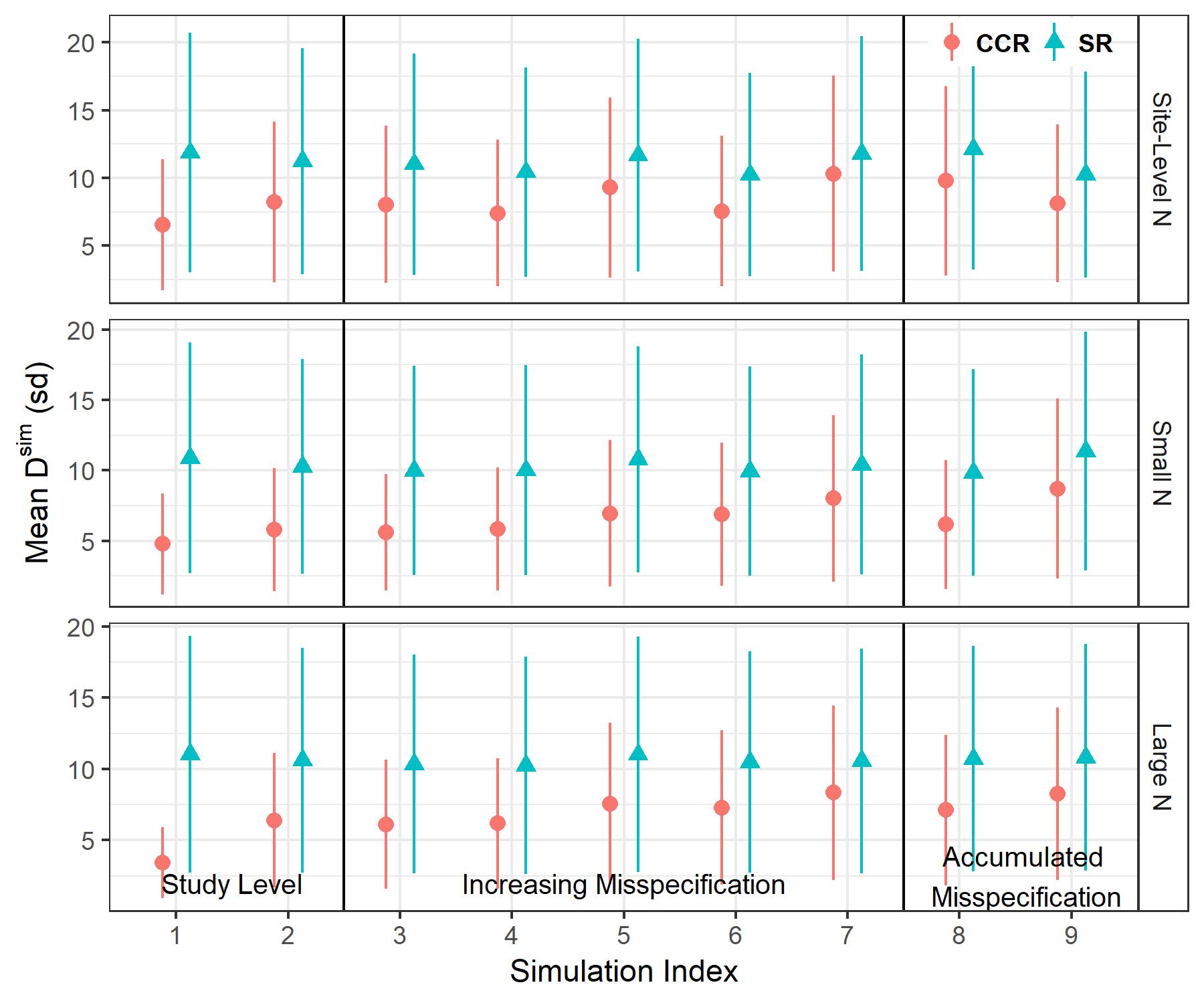


Figure A1 presents the mean simulated imbalance ($D^{sim}$) for CCR and SR across the full range of simulations. In general, $D_{SR}^{sim}$ values remain relatively constant as misspecification increases, but $D_{CCR}^{sim}$ values increase as misspecification increases. The difference between these measures generally decreases as misspecification increases, but CCR still outperforms SR even for large levels of misspecification.
